# Greater reductions in blood flow after anti-angiogenic treatment in non-small cell lung cancer patients are associated with shorter progression-free survival

**DOI:** 10.1038/s41598-021-86405-w

**Published:** 2021-03-24

**Authors:** Daisuke Katayama, Masahiro Yanagawa, Keiko Matsunaga, Hiroshi Watabe, Tadashi Watabe, Hiroki Kato, Takashi Kijima, Yoshito Takeda, Atsushi Kumanogoh, Eku Shimosegawa, Jun Hatazawa, Noriyuki Tomiyama

**Affiliations:** 1 Department of Nuclear Medicine and Tracer Kinetics, Osaka University Graduate School of Medicine, 2-2 Yamadaoka, Suita City, Osaka 565-0871 Japan; 2 Department of Radiology, Osaka University Graduate School of Medicine, 2-2 Yamadaoka, Suita City, Osaka 565-0871 Japan; 3 Department of Molecular Imaging in Medicine, Osaka University Graduate School of Medicine, 2-2 Yamadaoka, Suita City, Osaka 565-0871 Japan; 4 Division of Radiation Protection and Safety Control, Cyclotron and Radioisotope Center, Tohoku University, 6-3 Aoba, Aramaki, Aoba-ku, Sendai, Miyagi 980-8578 Japan; 5 Department of Respiratory Medicine and Hematology, Hyogo College of Medicine, 1-1, Mukogawa-cho, Nishinomiya, Hyogo 663-8501 Japan; 6 Department of Respiratory Medicine and Clinical Immunology, Osaka University Graduate School of Medicine, 2-2 Yamadaoka, Suita City, Osaka 565-0871 Japan; 7 Joint Research Division for Quantum Cancer Therapy, Research Center for Nuclear Physics, Osaka University, 2-1 Yamadaoka, Suita City, Osaka 565-0871 Japan

**Keywords:** Cancer, Lung cancer, Medical research

## Abstract

To evaluate tumor blood flow using ^15^O-water positron emission tomography (PET) in patients with non-small cell lung cancer (NSCLC) before and after chemotherapy with bevacizumab, and to investigate the effects of bevacizumab on tumor blood flow changes and progression-free survival (PFS). Twelve patients with NSCLC were enrolled. Six patients underwent chemotherapy with bevacizumab and the other six without bevacizumab. ^15^O-water dynamic PET scans were performed within 1 week before the start of chemotherapy and within 1 week after the first day of chemotherapy. Tumor blood flow was analyzed quantitatively using a single one-tissue compartment model with the correction of pulmonary circulation blood volume and arterial blood volume via an image-derived input function. In the bevacizumab group, mean tumor blood flow was statistically significantly reduced post-chemotherapy (pre-chemotherapy 0.27 ± 0.14 mL/cm^3^/min, post-chemotherapy 0.18 ± 0.12 mL/cm^3^/min). In the no bevacizumab group, there was no significant difference between mean tumor perfusion pre-chemotherapy (0.42 ± 0.42 mL/cm^3^/min) and post-chemotherapy (0.40 ± 0.27 mL/cm^3^/min). In the bevacizumab group, there was a positive correlation between the blood flow ratio (tumor blood flow post-chemotherapy/tumor blood flow pre-chemotherapy) and PFS (correlation coefficient 0.94). Mean tumor blood flow decreases after bevacizumab administration and was positively correlated with longer PFS.

## Introduction

Vascular endothelial growth factor (VEGF) is a major contributor to angiogenesis, which plays several important roles in local tumor progression and metastatic growth. Overexpression of VEGF has been observed in a variety of cancers including non-small cell lung cancer (NSCLC). Bevacizumab is a monoclonal anti-VEGF antibody that inhibits angiogenesis by preventing circulating VEGF from binding to its receptors^1^. The addition of bevacizumab to cytotoxic chemotherapy improves overall survival and progression-free survival (PFS) and is an accepted component of care for NSCLC^2^. Notably, however, the mechanism by which the combination of bevacizumab and cytotoxic chemotherapy improves survival in cancer patients remains unclear.

Positron emission tomography (PET) with ^15^O-labeled water is an established method for measuring tissue blood flow quantitatively. ^15^O-water is an ideal tracer for quantifying blood flow because it is distributed to tissue freely and cannot be metabolized. Compared to other PET tracers such as ^82^Rb, the correlation between ^15^O-water-derived values and actual blood flow is very high^3^. Additionally, ^15^O-water is a safe tracer. Since ^15^O-water is chemically the same as plain water, it does not cause allergic reactions or kidney injury, compared to iodine contrast agents. Moreover, in patients with renal failure, there is no risk of nephrogenic systemic fibrosis unlike when using gadolinium contrast agents. Previously, ^15^O-water PET was an invasive examination because it required continuous arterial blood sampling during the scan to obtain the input function. The image-derived input function, which is a non-invasive and accurate alternative to arterial sampling, is commonly used now^4^. PET using ^15^O-water is regarded as the gold standard for brain perfusion and it is now used in other areas such as myocardial perfusion and tumor perfusion^5,6^.

Imaging is commonly and widely used as a non-invasive procedure to evaluate treatment responses. The Response Evaluation Criteria in Solid Tumors (RECIST) are widely used to evaluate treatment responses, but in many cases, the criteria are not appropriate for predicting the outcomes of novel molecularly targeted cancer treatments because they are based on the reduction rate of morphological size^7^. PET facilitates the visualization of metabolic activity in tumors, resulting in more informative evaluations of treatment responses after chemotherapy. ^15^O-water PET enables the measurement of tumor perfusion defined as blood flow (mL/min) per cm^3^. We hypothesized that when assessing treatment responses to bevacizumab it may be possible to determine therapeutic effects more accurately by evaluating changes in tumor blood flow than by evaluating changes in tumor size identified via computed tomography (CT).

The purpose of the current prospective study was to evaluate tumor blood flow using ^15^O-water PET before and after chemotherapy with or without bevacizumab in patients with NSCLC and to investigate the effects of bevacizumab on tumor blood flow and PFS.

## Materials and methods

### Patients

**Figure 1:**
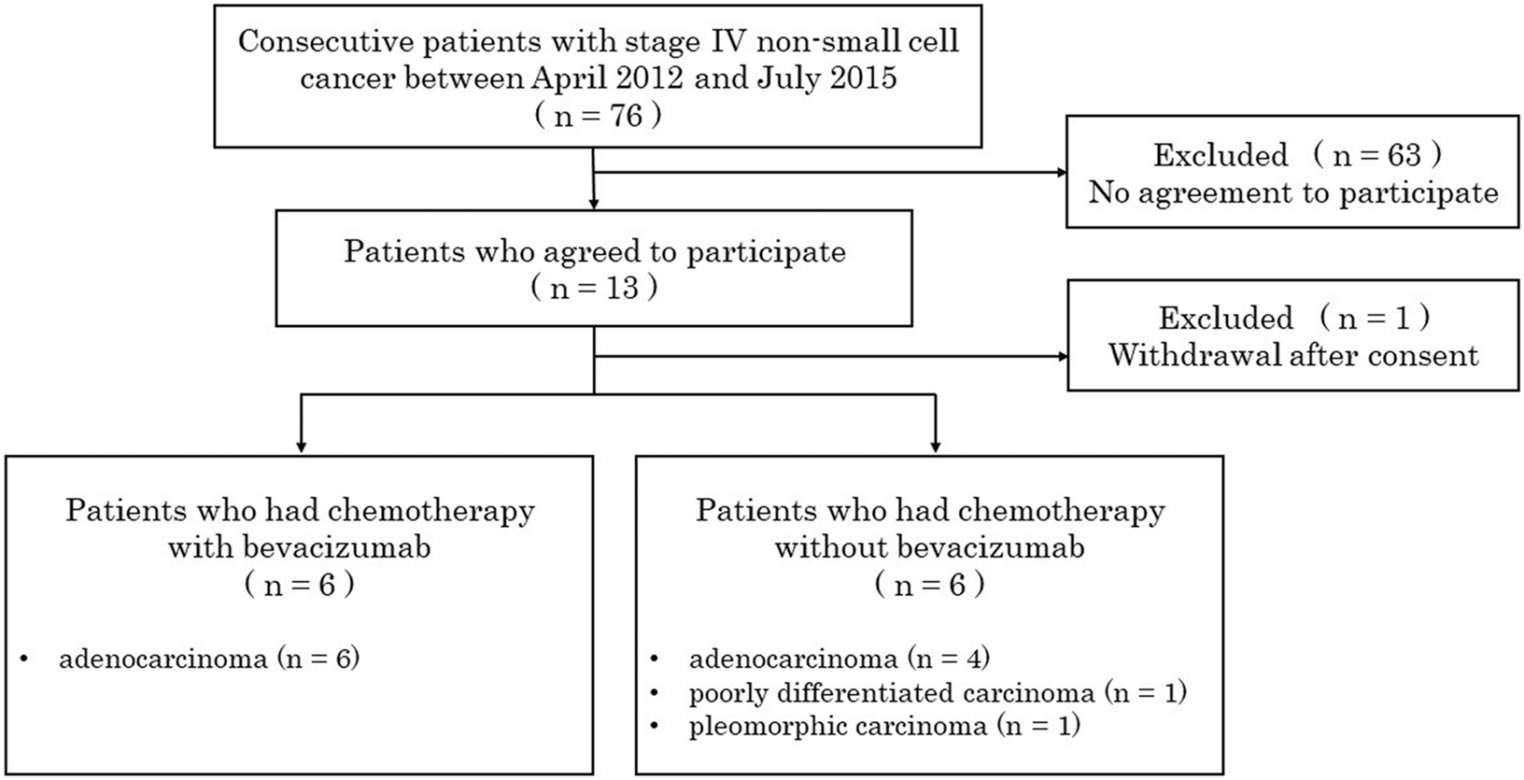
Study flowchart.

Between April 2012 and July 2015 patients with stage IV NSCLC who were scheduled to undergo chemotherapy at the Osaka University Hospital in Osaka, Japan were recruited by respiratory physicians before the start of the treatment. Of the 76 patients encountered during the study period, 13 agreed to participate in the study (Fig. 1). One patient withdrew from participation after providing consent. The final sample of subjects included 8 men and 4 women, with a mean age of 60 years (range 42–73 years). Ten patients had adenocarcinoma, 1 had pleomorphic carcinoma, and 1 had poorly differentiated carcinoma. The patient characteristics are summarized in Table 1. ^15^O-water PET is not covered by insurance in Japan. Approval from the internal Ethics Review Board was obtained before the initiation of the study. Informed consent was obtained from each patient.

**Table 1:** Patient characteristics.

Patient no	Sex	Age	Histological type	Chemotherapy regimen
**Chemotherapy with bevacizumab**				
1	M	62	Adenocarcinoma	CBDCA + PTX + BEV
2	F	67	Adenocarcinoma	CBDCA + PTX + BEV
3	M	62	Adenocarcinoma	CBDCA + PTX + BEV
4	M	65	Adenocarcinoma	CBDCA + PTX + BEV
5	F	42	Adenocarcinoma	CBDCA + PEM + BEV
6	M	73	Adenocarcinoma	CBDCA + PEM + BEV
**Chemotherapy without bevacizumab**				
7	M	46	Adenocarcinoma	CBDCA + PEM
8	M	61	Adenocarcinoma	CBDCA + PEM
9	M	64	Poorly differentiated carcinoma	CBDCA + PTX
10	F	42	Pleomorphic carcinoma	CBDCA + PTX
11	M	68	Adenocarcinoma	CDDP + PEM
12	M	71	Adenocarcinoma	CBDCA + PEM

*M* male; *F* female; *CBDCA* carboplatin; *PTX* paclitaxel; *BEV* bevacizumab; *PEM*, pemetrexed; *CDDP*, cisplatin.

### Chemotherapy

Initial chemotherapy was administered at the hospital to all the patients in the study. The chemotherapy regimens—including the use of bevacizumab—depended on the respiratory physicians treating each patient. Bevacizumab was generally prescribed with only a few exclusion criteria; squamous cell carcinoma and lesions with hemorrhagic changes or bloody sputum, which suggests hypervascularity, were excluded. In elderly patients with a history of cardiovascular events or comorbidities such as hypertension or renal dysfunction, the prescription of bevacizumab was also carefully assessed for each patient individually. Six patients (4 men and 2 women, mean age 62 ± 10 years) underwent chemotherapy that included bevacizumab, and the other 6 (4 men and 2 women, mean age 59 ± 11 years) had chemotherapy without bevacizumab. All patients in the bevacizumab group were diagnosed with adenocarcinoma histologically. Four of them were administered bevacizumab with carboplatin and paclitaxel, and two were administered bevacizumab with carboplatin and pemetrexed. The histological types in the no bevacizumab group included 4 adenocarcinomas, 1 pleomorphic carcinoma, and 1 poorly differentiated carcinoma. The chemotherapy regimens are shown in Table 1. The bevacizumab dose was 15 mg/kg of body weight. The doses of the other anticancer agents were carboplatin AUC 6, paclitaxel 200 mg/m^2^, pemetrexed 500 mg/m^2^, and cisplatin 80 mg/m^2^. In most regimens, all drugs were administered on day 1, and in all regimens, the next administration was 3 weeks thereafter.

### PET/CT imaging protocol

^15^O-water dynamic PET scans were obtained as a prospective study designed to evaluate tumor blood flow before and after bevacizumab administration. Baseline PET was performed within 1 week before the start of chemotherapy (mean 3.8 days, range 1–6 days). Post-chemotherapy PET was performed within 1 week after the first day of administration (mean 1.7 days, range 1–6 days). PET imaging was acquired on a SET-3000 GCT/X scanner (Shimadzu Corp., Kyoto, Japan). This scanner has an axial field of view of 26 cm, divided into 99 contiguous planes. The intrinsic spatial resolution is 3.5 mm full width at half maximum (FWHM) in-plane, and 4.2 mm FWHM axially.

Patients were positioned supine in the scanner bed with both the tumor and the aortic arch or heart in the center of the axial field of view. For attenuation correction, a 5-min transmission scan was performed using a ^137^Cs point source. After that transmission scan, a 10-min list mode scan was started simultaneously with an intravenous injection of 185 MBq of ^15^O-water (total amount 18.5 mL, injection speed 0.5 mL/s). The emission scan was reconstructed into 22 frames (1 frame × 10 s, 8 frames × 5 s, 4 frames × 10 s, 2 frames × 15 s, 3 frames × 20 s, 2 frames × 30 s, and 6 frames × 60 s) using the two-dimensional dynamic row-action maximum-likelihood algorithm after three-dimensional Gaussian smoothing with a 6-mm FWHM. The voxel size was 4.7 × 4.7 × 2.6 mm.

After the emission scan, a reference CT was performed. The CT acquisition parameters were breath-hold at shallow inspiration, from the apex of the lung to the base of the lung, no intravenous media, 120 kVp and 50 effective mAs, 52 slices, and 5.0-mm slice thickness. Clinically baseline CT scanning was performed up to 1 month before the start of chemotherapy. Follow-up CT scans were performed after 2 courses of chemotherapy and every 2–4 months thereafter. Brain magnetic resonance imaging and bone scintigraphy were performed as needed. PFS was defined as the period from treatment start until progression was seen on radiological examination. Progression included progression of the primary tumor, local recurrence, and the appearance of metastases.

### Quantitative analysis for tumor blood flow

Tumor perfusion was calculated via the following equation (Eq. 1), using a single one-tissue compartment model with the correction of pulmonary circulation blood volume and arterial blood volume^4^.

1 $$CPET\left( {\text{t}} \right) = (1 - VA - VV) \cdot F \cdot CA\left( {\text{t}} \right) \otimes e - (F/VT)\;t + VACA\left( {\text{t}} \right) + VVCV\left( {\text{t}} \right)$$

where C_PET_ (t) = tracer concentration in tissue (Bq/cm^3^), *C*_*A*_(t) = tracer concentration in artery (Bq/cm^3^), *C*_*V*_(t) = tracer concentration in the right ventricular cavity (Bq/cm^3^), *F* = blood flow (mL/cm^3^/min), *V*_*T*_ = partition coefficient of water, *V*_*A*_ = arterial blood volume, and *V*_*V*_ = pulmonary circulation blood volume.

The image-derived input function was used. Volumes of interest (VOIs) with a diameter of 1 cm were drawn over the ascending aorta in approximately 10 consecutive image planes of the frame in which the first pass of the bolus was best visualized. Projection of the resulting VOI onto all image frames yielded the arterial time-activity curve or image-derived input function *C*_*A*_(t). Applying a similar approach to the right ventricular cavity provided a time-activity curve for the pulmonary circulation *C*_*V*_(t). Parametric images of perfusion were generated using the basis function method^8^. In the present study, 300 logarithmically spaced precomputed basis functions with *F/V*_*T*_ values ranging from 0.0 to 0.4 min^−1^ were used. One radiologist placed manually each VOI over the tumor with reference to the CT image, making the VOI as large as possible to minimize the effects of inhomogeneity and was projected onto the parametric perfusion images using PMOD 3.6 (PMOD Technologies, LLC, Zürich, Switzerland). All tumors did not show obvious necrotic findings such as cavity formation on CT images, therefore all tumors were analyzed for perfusion in their entire area. The averaged tumor perfusion over the VOI in the parametric perfusion images was used for statistical analyses.

### Statistical analysis

Tumor blood flow before and after chemotherapy were compared in the bevacizumab group and the no bevacizumab group. In the bevacizumab group, tumor blood changes and PFS were examined. All statistical analyses were performed using commercially available software (MedCalc version 19.2.1, Frank Schoonjans, Mariakerke, Belgium). Differences in mean tumor blood flow before and after chemotherapy in each group were analyzed using the Wilcoxon test. In the bevacizumab group, regression analysis was performed on the blood flow ratio before and after chemotherapy and PFS. *p* < 0.05 was considered statistically significant.

### Ethics approval and consent to participate

All procedures performed were approved by the ethical committee of Osaka University Hospital (No.11109) and were conducted in accordance with the principles of the Declaration of Helsinki. Informed consent was obtained from all individual participants included in the study.

## Results

**Figure 2:**
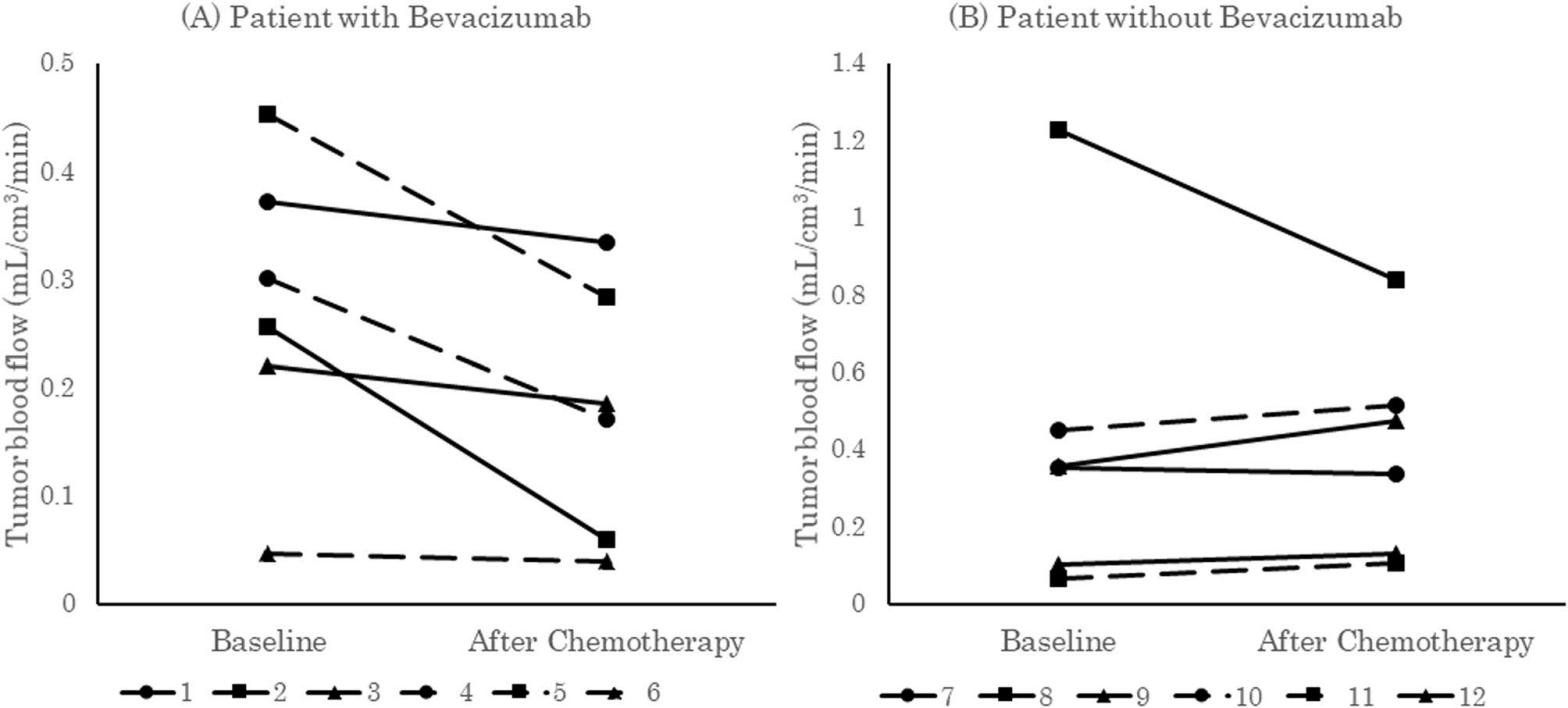
Tumor blood flow before and after chemotherapy in each patient. (**A**) Patients who underwent chemotherapy with bevacizumab. There was a statistically significant decrease in blood flow. (**B**) Patients who underwent chemotherapy without bevacizumab. There was no statistically significant change in blood flow.

In the bevacizumab group, mean tumor perfusion was statistically significantly reduced after chemotherapy (pre-chemotherapy 0.27 ± 0.14 mL/cm^3^/min, post-chemotherapy 0.18 ± 0.12 mL/cm^3^/min). In the no bevacizumab group, there was no significant difference between mean tumor perfusion pre-chemotherapy (0.42 ± 0.42 mL/cm^3^/min) and post-chemotherapy (0.40 ± 0.27 mL/cm^3^/min) (Fig. 2). Table 2 shows the tumor volume and the tumor blood flow measurements in each patient. The VOI sizes were on average 47.4 cm^3^ (range 1.89–140 cm^3^) in the bevacizumab group and 51.3 cm^3^ (range 4.20–137 cm^3^) in the no bevacizumab group. There was no correlation between tumor volume and tumor blood flow before chemotherapy.

**Table 2:** Quantitative tumor blood flow values in each patient.

Patient no	Tumor volume	F baseline	F after treatment	F_after_/F_baseline_
**Chemotherapy with bevacizumab**				
1	42.4	0.371	0.334	0.899
2	6.63	0.256	0.059	0.231
3	1.89	0.221	0.185	0.838
4	89.5	0.301	0.171	0.567
5	140	0.452	0.284	0.628
6	3.66	0.047	0.039	0.824
**Chemotherapy without bevacizumab**				
7	8.51	0.351	0.335	0.956
8	54.0	1.225	0.838	0.684
9	4.97	0.100	0.130	1.292
10	99.2	0.449	0.514	1.145
11	4.20	0.063	0.104	1.648
12	137	0.358	0.474	1.322

F_after_/F_baseline_ is the ratio of tumor blood flow after treatment to tumor blood flow at baseline.

Tumor volume (cm^3^), F: tumour blood flow (mL/cm^3^/min).

**Figure 3:**
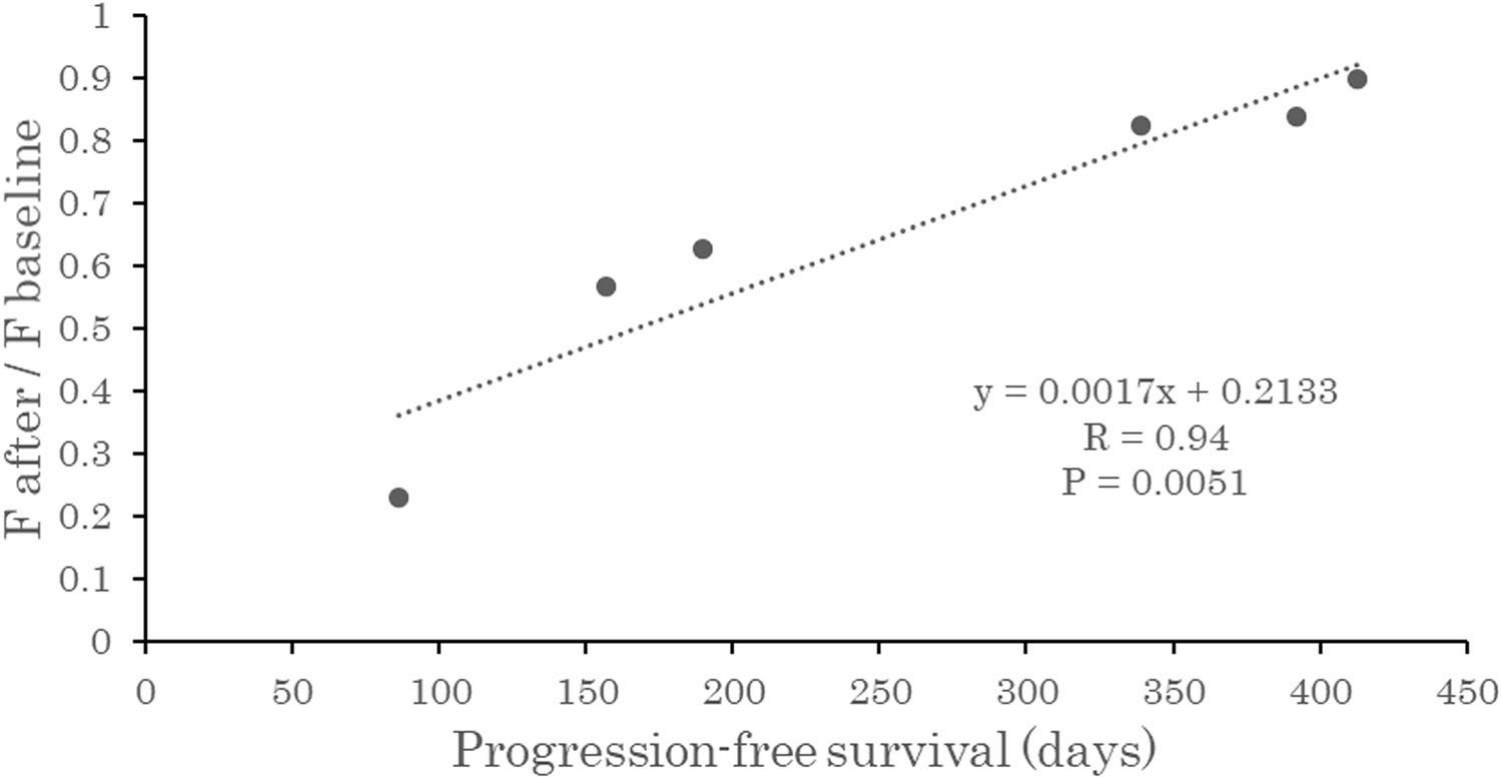
Relationships between change in tumor blood flow and progression-free survival in patients who underwent chemotherapy with bevacizumab. The vertical line shows the ratio of tumor perfusion at the baseline to tumor perfusion after administration, and the horizontal one shows progression-free survival. A substantial reduction in tumor perfusion after bevacizumab administration was associated with shorter time to progression.

**Figure 4:**
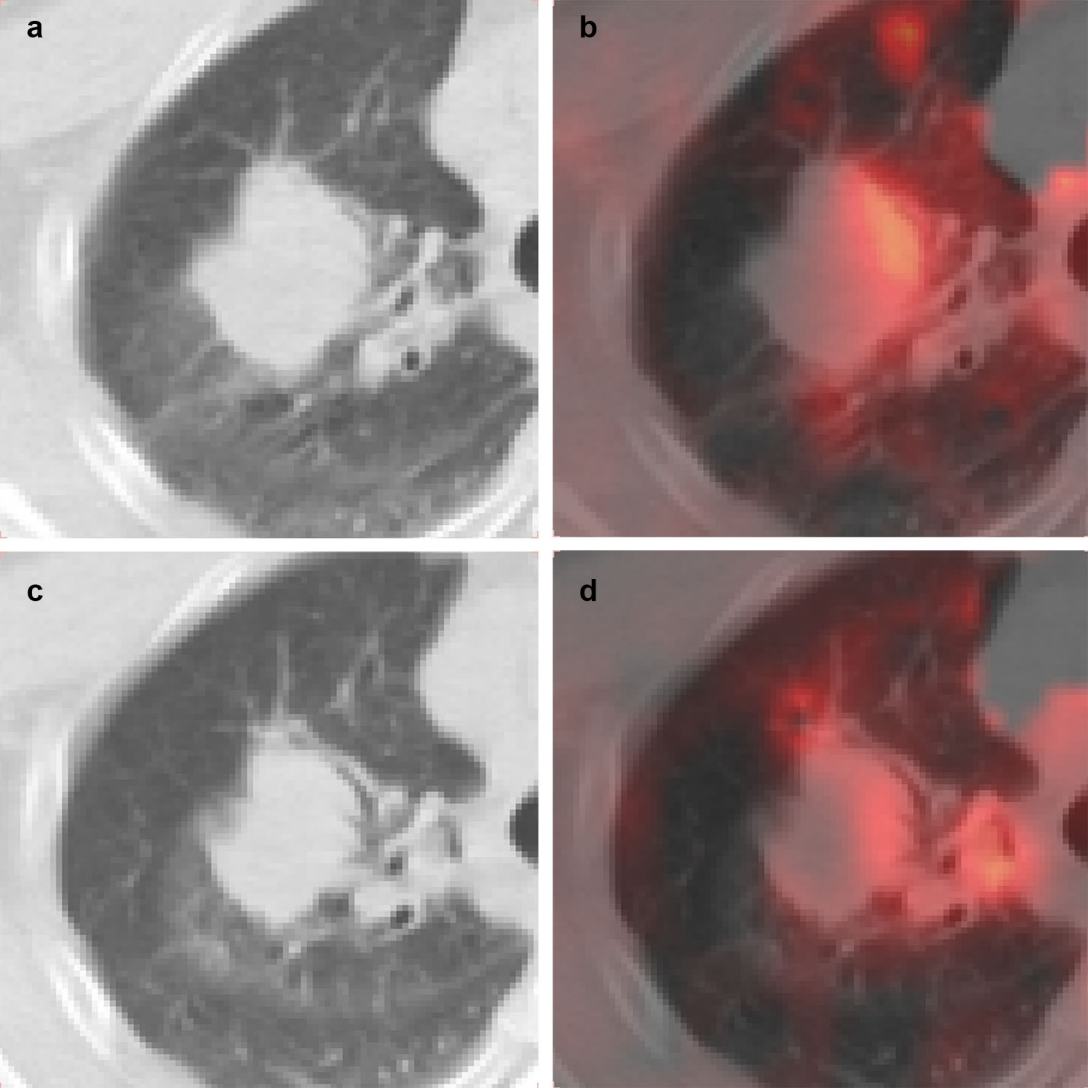
^15^O-water PET image of a 62-year-old man with adenocarcinoma treated with bevacizumab (patient no.1). (**a,b**) CT and fusion image of CT and parametric image of blood flow at baseline. (**c,d**) CT and fusion image after chemotherapy. The CT showed a 4.4 cm large mass in the right lung. Tumor blood flow was 0.371 mL/mL/min before treatment and slightly decreased to 0.334 mL/mL/min after chemotherapy (blood flow ratio: 0.90). He had no tumor progression for 1 year and 2 months.

**Figure 5:**
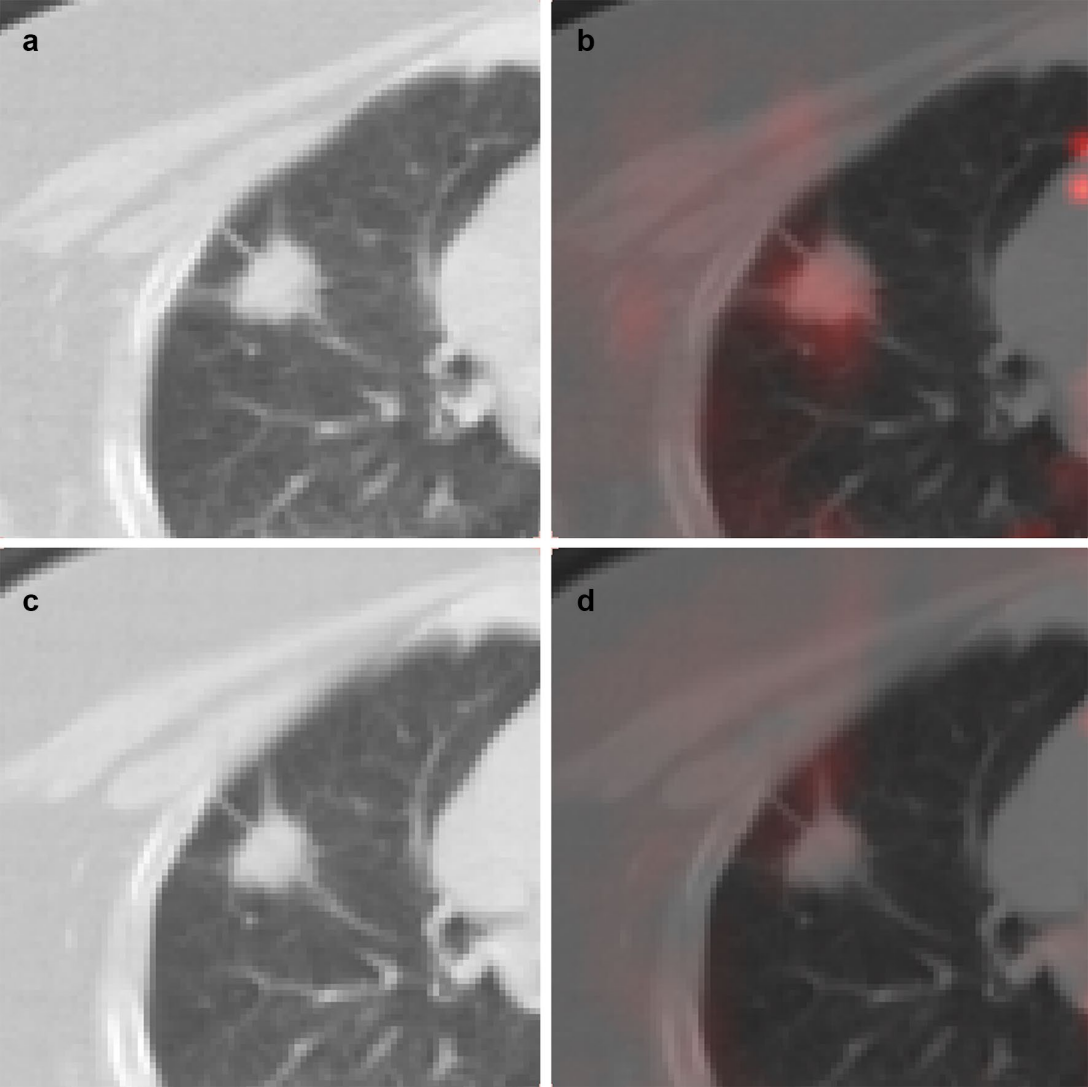
^15^O-water positron emission tomography of a 67-year-old woman with adenocarcinoma treated with bevacizumab (patient no. 2). (**a,b**) CT and fusion image of CT and parametric image of blood flow at baseline. (**c,d**) CT and fusion image after chemotherapy. CT depicted a 2.6-cm nodule in the right lung. Tumor blood flow was markedly reduced, from 0.256 mL/cm^3^/min to 0.059 mL/cm^3^/min (blood flow ratio 0.23). Three months later the tumor exhibited progression.

Follow-up study showed that all patients had tumor progression eventually. In the bevacizumab group, five of the six patients had local progression, and the remaining one had progression of pleural dissemination. In the no bevacizumab group, two of the six patients had local progression, and the other four patients had progression of metastatic or disseminated lesions. PFS was not significantly correlated with tumor blood flow before or after chemotherapy in either group. In the bevacizumab group, the rate of decline in tumor blood flow varied markedly in different patients. There was a positive correlation between the blood flow ratio (post-chemotherapy tumor blood flow/pre-chemotherapy tumor blood flow) and PFS (correlation coefficient 0.94), yielding a regression equation of y = 0.2729 + 0.001616x (*p* = 0.005) (Fig. 3). A smaller blood flow ratio after chemotherapy was associated with a shorter time to tumor progression (Fig. 4) (Fig. 5). In the no bevacizumab group, there was no significant correlation between the blood flow ratio and PFS (correlation coefficient -0.15, *p* = 0.77).

## Discussion

In the present study, bevacizumab was associated with significantly reduced tumor blood flow 1–2 days after chemotherapy. Notably, however, follow-up investigations revealed that this effect was associated with rapid tumor progression. Patients with only a slight change in blood flow tended to exhibit longer PFS. ^15^O-water PET was an optimal and non-invasive method for assessing treatment responses to chemotherapy regimens including bevacizumab in patients with NSCLC. The vascular normalization theory proposed by Jain^9^ may explain this phenomenon. Unlike physiological angiogenesis processes such as wound healing, tumor angiogenesis continues abnormally while the tumor is growing because the tumor requires a vascular supply to provide essential nutrients and oxygen. Tumor vessels are often tortuous, disorganized, and highly permeable, resulting in high interstitial pressure and reduced blood perfusion and oxygenation. Tumor cells can adapt to insufficient blood supply and hypoxia, but drug delivery is inhibited and its efficacy is reduced. Excessive antiangiogenic therapy may lead to reduced tumor blood flow and result in hypoxia and acidosis, which promote tumor progression^10^. Moderate anti-VEGF therapy may lead to ‘vascular normalization’, which is characterized by an attenuation of hyperpermeability, increased vascular pericyte coverage, a more normal basement membrane, and a resultant reduction in tumor hypoxia and interstitial fluid pressure. As a result, drug delivery of cytotoxic anticancer agents is improved, and consequently, chemotherapy in combination with anti-VEGF drugs improves survival.

In the current study, a decline in tumor blood flow after bevacizumab administration was observed in all patients, but patients with greater reductions in tumor blood flow exhibited tumor progression within shorter periods. It may be that a greater reduction of tumor perfusion reflects greater pruning of vessels, which leads to hypoxia and acidosis in the tumor. Heist et al.^11^ reported that reduced blood perfusion after bevacizumab administration as determined via CT was associated with shorter overall survival in NSCLC patients, which is consistent with the results of our study. They assessed tumor perfusion before bevacizumab administration and 14 days thereafter. In our study, the assessment of tumor perfusion was performed 1–2 days after bevacizumab administration. This suggests that it may be possible to predict the effects of chemotherapy just days after the administration of bevacizumab using ^15^O-water PET. If bevacizumab is found to be insufficiently effective at an early stage, switching to another anticancer drug could be considered earlier. In addition, ^15^O-water PET can be performed at a lower radiation dose than perfusion CT (^15^O-water PET, 0.37 mSv vs. perfusion CT, 3.5–6.5 mSv)^12^.

Tumor blood flow is still not well understood, so it is necessary to clarify how tumor blood flow before treatment and changes in tumor blood flow after treatment affect the efficacy of anticancer therapy and prognoses. Accordingly, a larger number of cases needs to be examined. Tumor blood flow is associated with tumor hypoxia, which is associated with resistance to chemotherapy. An explanation for why the extent of the reduction in tumor blood flow is associated with the response to chemotherapy may be correlated with the altered hypoxic region of the tumor. Further insights may be obtained by combining hypoxic imaging with a radiolabeled tracer such as ^18^F-fluoromisonidazole^13^. In bevacizumab group of the present study, all 6 patients presented tumor progression. Five of 6 patients showed regrowth of the lesions where tumor blood flow was evaluated. Therefore, each PFS of 5 patients was consistent to the time to progression of the site where tumor blood flow was measured. In the remaining one patient (patient no. 3), local regrowth was not observed for a long time in the lesion where tumor blood flow was measured, but a pleural dissemination eventually progressed. Therefore, in the present study, PFS was correlated with the time to progression of the site where tumor blood flow was measured. Although ^15^O-water PET in NSCLC patients treated with bevacizumab might be relevant only to the prediction for the time to progression of the site where blood flow is measured, further study using a large cohort is needed to examine whether blood flow changes in the primary tumor can predict not only the time to progression of the local regrowth but also the prognosis of metastatic lesions.

The current study had several limitations. First, in this analysis, only the largest lesions in the lung were evaluated to measure tumor blood flow. Other lesions such as lymph node, bone and brain metastases were not evaluated in this study. The next limitation is the small number of participants, which resulted in low statistical power. We asked 76 consecutive lung cancer patients to participate in the study, but only 13 agreed to participate. It is assumed that many of the patients did not want to participate because of the need for extra hospital visits and because there was no direct benefit to the patients themselves from participating in the study. Moreover, several biases may have occurred due to patient selection, chemotherapy regimen, and tumor histology because of the following reasons. This study was conducted at a single facility, and the bevacizumab prescription was not randomized, which may have led to a bias between the bevacizumab group and the no bevacizumab group. There were also some technical limitations. The parametric images obtained are prone to noise, which slightly reduces the reliability of the quantification. Each VOI was manually placed over a pulmonary nodule without margins, making it as large as possible in an effort to minimize the effects of inhomogeneity. Therefore, blood flow may be underestimated due to partial volume effects at the border of the tumor and normal lung tissue, especially in small-sized lesions.

In conclusion, in the current study, mean tumor blood flow diminished within 1–2 days after bevacizumab administration in NSCLC patients, and greater reductions in blood flow were associated with shorter PFS.

## Data Availability

The datasets generated during and/or analyzed during the current study are available from the corresponding author on reasonable request.
